# Age-period-cohort analysis and prediction of tuberculosis trends in China—based on the Global Burden of Disease 2021 data

**DOI:** 10.3389/fpubh.2025.1512514

**Published:** 2025-02-14

**Authors:** Zhifei Chen, Xiaodie Chen, Minli Chang, Dongmei Lu, Liping Zhang, Yanling Zheng

**Affiliations:** ^1^College of Medical Engineering and Technology, Xinjiang Medical University, Urumqi, Xinjiang, China; ^2^School of Public Health, Xinjiang Medical University, Urumqi, Xinjiang, China; ^3^Center of Pulmonary and Critical Care Medicine, People’s Hospital of Xinjiang Uygur Autonomous Region, Urumqi, Xinjiang, China; ^4^Institute of Medical Engineering Interdisciplinary Research, Xinjiang Medical University, Urumqi, Xinjiang, China

**Keywords:** tuberculosis, Joinpoint regression model, APC model, BAPC model, prediction

## Abstract

**Background:**

This study explores the epidemic trend of tuberculosis (TB) in China from 1990 to 2021, analyzes its relationship with age-period-cohort factors from 1992 to 2021, and predicts the development trend of TB in China from 2022 to 2046.

**Methods:**

Annual Percent Change (APC), Average Annual Percent Change (AAPC) and 95% confidence interval (*CI*) were calculated by Joinpoint regression model to describe the epidemic trend of TB in China. The Age-Period-Cohort (APC) model was used to explore the effects of age, period and cohort effects on the trend of TB incidence rate, mortality and DALYs rate. APC model and Bayesian Age-Period-Cohort (BAPC) model were used to predict the epidemic trend of TB in China from 2022 to 2046.

**Results:**

In 2021, it is estimated that there are 617,700 incidence cases of TB, 37,300 deaths and 1,375,500 DALYs cases in China, and the corresponding number of male cases is higher than that of female cases. From 1990 to 2021, the number of TB incidence, deaths and DALYs, as well as ASIR, ASDR and ASR of DALYs in China decreased year by year. The AAPC of ASIR, ASDR and ASR of DALYs were −3.33, −7.28% and −6.77%, respectively, all *p* < 0.05, indicating that the overall trend showed a significant decrease. With the increase of age, the incidence rate of TB first decreased, then increased and then decreased, while the mortality and DALYs rate first decreased rapidly and then decreased slowly, and both reached the highest value in the <5 years age group. The period effect showed that the risk of the total population, male and female population decreased overall. The cohort effect showed that the risk of TB incidence rate, mortality and DALYs rate decreased significantly. The ASIR, ASDR and ASR of DALYs of TB in China are predicted to decrease year by year from 2022 to 2046. The BAPC model predicts that the number of incidence, deaths and DALYs will reach 177,100,077,000 and 181,700 in 2046, respectively.

**Conclusion:**

The APC model shows that the earlier the contemporary people are born, the older the age, the higher the risk of disease. APC and BAPC models predict that the ASR of TB in China will decrease year by year, but men and the older adult are still at high risk of TB burden in China. It is recommended to strengthen the screening of TB patients in key populations, especially in the older adult.

## 1 Introduction

Tuberculosis (TB) is an ancient chronic infectious disease, once known as “white plague” and “tuberculosis.” It is caused by *Mycobacterium tuberculosis* (MTB) and can affect a variety of organs, especially lung infection (1). It is mainly transmitted through the respiratory tract (2). It has the characteristics of long incubation period, early symptoms are not obvious, and easy to spread (3). Tuberculosis is considered to be a major public health problem worldwide, and it is one of the major infectious diseases that China has long focused on (4). According to the “2024 Global Tuberculosis Report” released by the World Health Organization (WHO) on October 29, 2024 (1), the estimated number of TB cases in China in 2023 is 741,000 (748,000 in 2022), and the incidence rate is 52 per 100,000 (53 per 100,000 in 2022). It is estimated that the number of TB new incidence cases in China still ranks third in the world, and there are still a large number of new cases in China (5, 6). Therefore, the task of TB prevention and control in China is still arduous. The Global Burden of Disease 2021 (GBD 2021) study comprehensively assessed and quantified the burden of TB-related diseases. A recent study revealed significant differences in TB burden among different genders, age groups and regions through a systematic analysis of the global TB burden, emphasizing the impact of socio-economic development levels on the TB burden (7). Therefore, countries at different levels of socio-economic development must take targeted measures according to their national conditions to solve the serious burden caused by TB. China’s population accounts for about one-fifth of the global population (8) and plays a key role in shaping global health outcomes. Previous studies (9) analyzed the TB burden of the older adult in China based on the GBD 2019 database, but there were limitations such as a narrow population range and no prediction of future TB burden.

Understanding the epidemic status and development trend of TB in the population and taking corresponding public health measures are essential for the prevention and control of TB in China (10, 11). At present, there are many related methodologies for studying the trend of disease burden. However, in recent years, Joinpoint regression model and Age-Period-Cohort (APC) model have been favored by a large number of scholars. These two models can well analyze the trend of disease epidemic and the influence of age, period and cohort effect on the trend (12, 13). Suixiang Wang et al. (14) used a combination of Joinpoint regression model and APC model to study the incidence of cervical cancer and ovarian cancer in women in Guangzhou, China. The results showed that the incidence of cervical cancer and ovarian cancer in women in Guangzhou, China showed a downward trend, the mortality rate increased with age, and the incidence and mortality decreased with the increase of birth cohort. In recent years, APC model and Bayesian Age-Period-Cohort (BAPC) model have been widely used in analyzing and predicting disease trends (15, 16). Haiping Gu et al. (17) predicted the disease burden of rheumatoid arthritis in China from 2022 to 2046 using the APC model and the BAPC model, indicating that the incidence of rheumatoid arthritis will continue to rise, but the mortality rate has been declining.

At present, there are few studies on the trend and prediction of TB burden in China by using Joinpoint regression model, APC model and BAPC model. In this study, Joinpoint regression model was used to analyze the trend of TB burden in China from 1990 to 2021. APC model was used to estimate the influence of age, period and cohort effect on the trend of TB burden in Chinese population, and to determine the high-risk group of TB in Chinese population. APC model and BAPC model were used to predict the trend of TB burden in China from 2022 to 2046. The research results can provide a theoretical reference for mastering the epidemic law of TB in China and better preventing and controlling the disease.

## 2 Materials and methods

### 2.1 Data sources

GBD 2021 database^1^ has been widely recognized by many epidemiological researchers for its ability to systematically and comprehensively collect and analyze epidemiological data. The database covers the standardized measures of incidence rate, mortality, disability-adjusted life years (DALYs) and other indicators for 371 diseases and injuries in 204 countries and regions during the period 1990–2021. The data retrieval strategy of this study includes: the disease is TB, the region is China, the gender category is divided into three categories: male, female and total population, and the age category is divided into 20 categories from <5 years, 5–9 years to 95+ years. The selected data types are numbers and ratios. The analysis focused on Age-Standardized Incidence Rate (ASIR), Age-Standardized Deaths Rate (ASDR) and Age-Standardized Rate of Disability-Adjusted Life Years (ASR of DALYs). The definition of TB was based on the revised codes of the ninth edition (ICD-9) and the tenth edition (ICD-10) of the International Classification of Diseases.

The prevalence trend of TB in China from 1990 to 2021 was evaluated by using ASIR, ASDR and ASR of DALYs of TB by gender and age. The GBD 2021 Chinese age-specific standardized population model matches these indicators. By introducing this method, the prevalence trends of ASIR, ASDR and ASR of DALYs of TB and the burden over time can be accurately compared. The reliability of GBD 2021 data has been confirmed by previous studies (18).

### 2.2 Joinpoint regression model

Determining changes in recent disease trends helps to analyze disease burden data. The Joinpoint regression model is often used to analyze the temporal characteristics of disease or injury distribution. It was first applied by Kim et al. to study the trend of cancer mortality, and then gradually applied by more and more researchers in the field of epidemiology (19). The model calculates the annual percentage change (APC), average annual percentage change (AAPC) and 95% confidence interval (95%*CI*) of ASIR, ASDR and ASR of DALYs of TB (20). The specific formula is as follows:


$$\ln\left(\gamma \right)=\alpha +\beta_{i}x+\varepsilon$$

$$APC\left(\%\right)=\left(e^{\beta 1}-1\right)\times 100$$

$$\mathrm{AAPC}\left(\%\right)=\left[\exp\frac{\sum \omega_{i}\beta_{i}}{\sum \omega_{i}}-1\right]\times 100$$

In the formulas, γ represents the rate, x is the year, α is the intercept, $\beta_{i}$ is the slope coefficient for each time segment, and $\omega_{i}$ is the number of years in each time segment.

The model uses a log-linear model to fit the data, scientifically divides the long-term trend line into several sub-cycles, and then uses the Monte Carlo permutation test and the modified Bayesian information criterion to optimize the model selection, minimize the mean square error, and determine the preferred number of connection points, location, and length of each sub-cycle, so as to evaluate the disease variation characteristics in different time intervals in more detail (21). The APC evaluates the changes over the set period, and the AAPC represents a composite indicator of overall changes over time. APC > 0 means year-on-year increase, otherwise it means decrease. When APC = AAPC, it indicates that there is no major turning point in the trend, and the overall trend is monotonous (22). Test level = 0.05 (bilateral).

### 2.3 Age-period-cohort model

APC model is often used to analyze and evaluate the impact of age, period and cohort effect on the trend of TB burden. In recent years, the APC model analysis method has gradually matured and has been widely used in disease trend analysis (10). Age effect refers to the effect of disease rate changing with age, which is one of the most important factors affecting the occurrence of disease. Period effect refers to the change of human factors affecting the disease rate of the population, such as the development of disease diagnosis technology, screening and early detection, disease definition and registration changes, treatment improvement, etc. These human factors may affect the period effect of disease rate in different periods. Cohort effect refers to the change of disease rate due to the different degree of exposure to risk factors in different age groups. Traditional statistical methods cannot eliminate the collinearity between these factors. The intrinsic estimator (IE) bypasses the traditional statistical methods and solves the problem that the parameters cannot be estimated (23). APC model is a linear model, the specific formula is as follows:


$$Y=\log\left(M\right)=\mu +\alpha age_{i}+\mathrm{\beta perio}d_{j}+\mathrm{\gamma cohor}t_{k}+\varepsilon$$

In the formula, Y represents the response variable, M represents the disease rate. α , β , and γ represent the coefficients of the age, period, and cohort effects in the APC model, μ represents the intercept of the model. ε represents the residual of the APC model, and age+cohort=period .

### 2.4 APC and BAPC prediction models

APC model and BAPC model were introduced to predict the development trend of disease burden. APC model is a parametric statistical model, which is often used to analyze the trend of chronic disease burden and predict the future change of disease burden (24). The APC model considers the effects of age, period, and cohort factors on the distribution of diseases. However, due to the linear relationship between these three factors, there may be cases where the parameter estimates are not unique. The BAPC model is a logarithmic linear Poisson model, which is often used to predict the trend of disease burden. It is a Bayesian version of the APC model. It uses both sample information and prior information to obtain a unique parameter estimate. The results are robust and reliable (25). The BAPC model uses the integrated nested Laplace approximation (INLA) to approximate the posterior edge distribution, thus avoiding the mixing and convergence problems and showing a relatively low error rate (26). Therefore, based on the APC model, the BAPC model is added to further predict the future development trend of TB in China.

### 2.5 Statistical methods

Excel 2021 is used to edit and process data, and dual data entry and validation methods are used to ensure the accuracy of the data. Joinpoint regression model was constructed using Joinpoint software (4.7.0.0). APC, AAPC and their 95% confidence intervals (*CI*) were calculated and the trend changes were analyzed. The APC model analyzes and evaluates the effects of age, period and cohort effects on TB epidemic trends by using the network analysis tool^2^ provided by the National Cancer Institute (NCI). The APC and BAPC prediction models were established using the ‘nordpred’ (version 1.1) and ‘BAPC’ (version 0.0.36) packages in the R software (4.4.1), respectively. The ‘nordpred’ package is commonly used to predict the disease burden, while the ‘BAPC’ package uses Bayesian inference to overcome the identifiability problem in the APC model. We use ‘ggplot 2’ (version 3.5.1) software package to create graphics, and use ‘ggsci’ (version 3.1.0) software package to create a scientific palette for data visualization. This study suggests that *p* < 0.05 was statistically significant.

## 3 Results

### 3.1 Descriptive analysis of the TB burden in China in 2021

**Figure 1:**
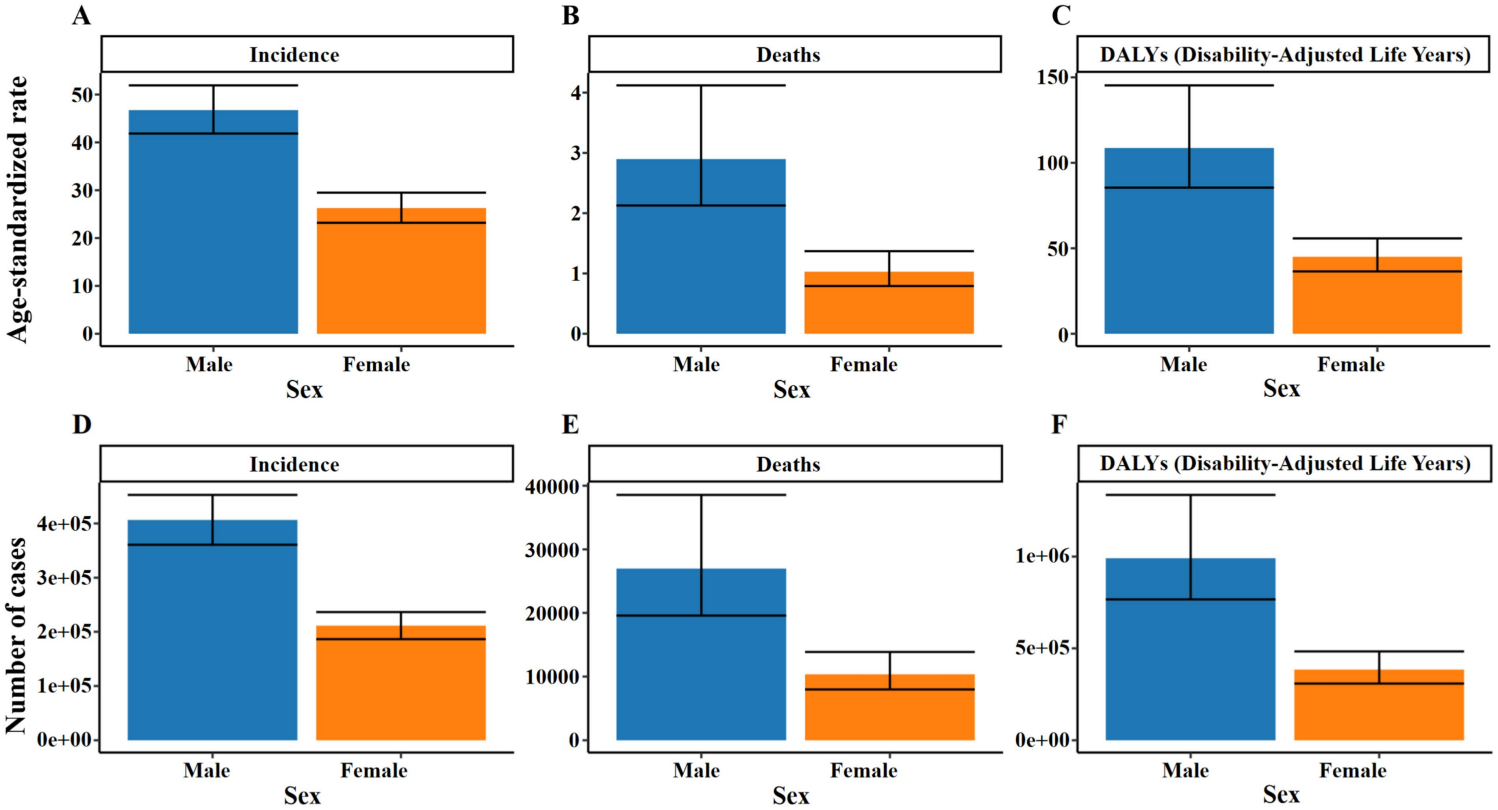
Number and ASR of TB incidence, mortality, and DALYs in China for 2021, categorized by gender. **(A)** ASIR. **(B)** ASDR. **(C)** ASR of DALYs. **(D)** Number of incidence cases. **(E)** Number of deaths cases. **(F)** Number of DALYs incidence cases.

The GBD 2021 database shows that there are an estimated 617,700 new TB cases in China in 2021, including 406,400 males (95%UI: 360,700 to 452,600) and 211,300 females (95%UI: 186,400 to 236,400). It is estimated that there were 37,300 deaths caused by TB, including 27,000 males (95%UI: 19,600 to 38,600) and 10,400 females (95%UI: 8,000 to 13,900). It is estimated that there were 1,375,500 DALYs cases caused by TB, including 991,200 males (95%UI: 767,300 to 1,335,600) and 384,300 females (95%UI: 308,400 to 483,200) (Figure 1).

**Figure 2:**
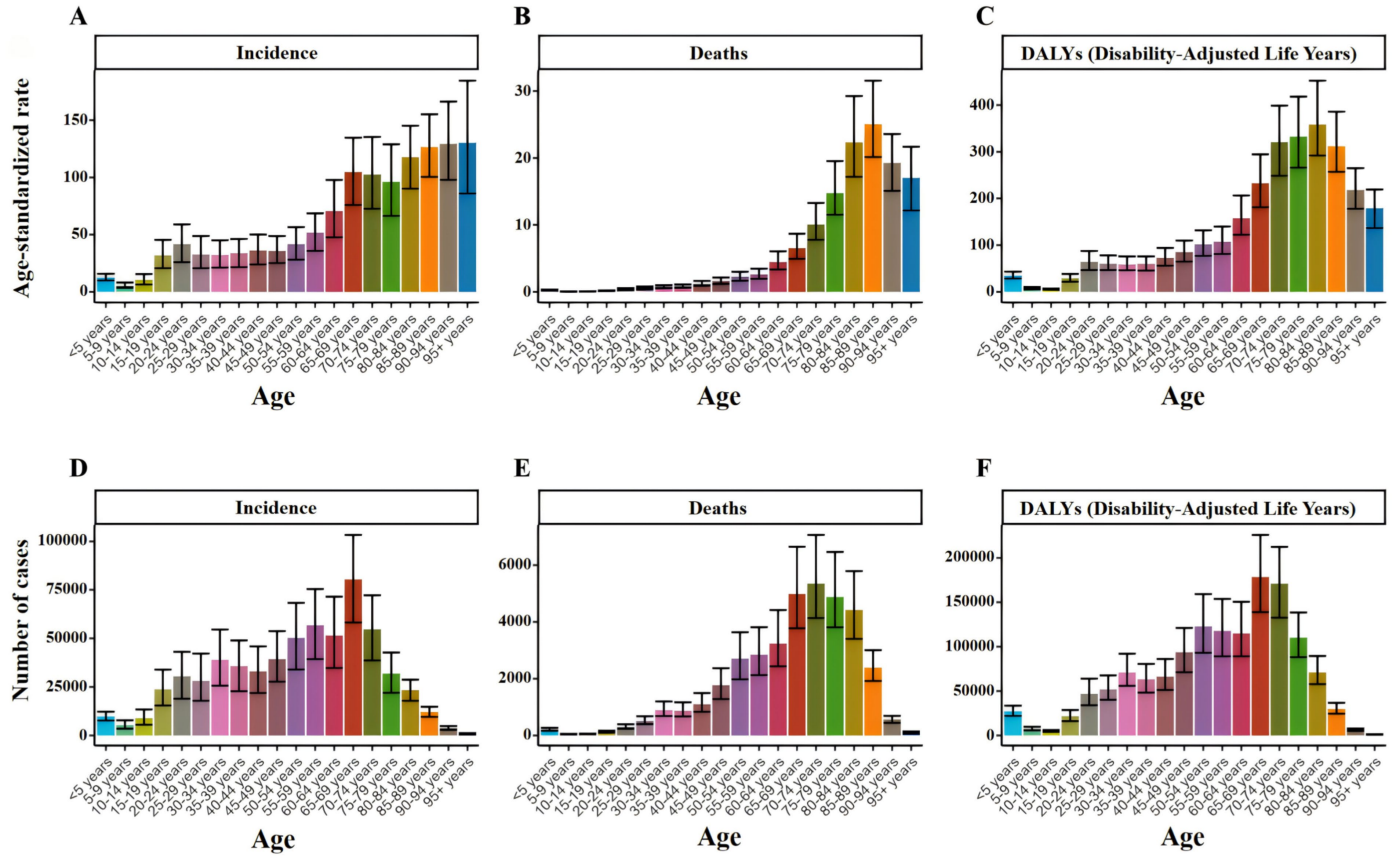
Number and ASR of TB incidence, mortality, and DALYs in China for 2021, categorized by age groups **(A)** ASIR. **(B)** ASDR. **(C)** ASR of DALYs. **(D)** Number of incidence cases. **(E)** Number of deaths cases. **(F)** Number of DALYs incidence cases.

In 2021, the ASIR of TB in China was 36.28 per 100,000 people, with 46.75 (95%UI: 41.84 to 51.91) for males and 26.27 (95%UI: 23.19 to 29.49) for females. The ASDR was 1.91 per 100,000 people, and 2.90 (95%UI: 2.13–4.12) for men, again higher than 1.03 (95% UI: 0.79–1.37) for women; the ASR of DALYs was 76.22 per 100,000 people, and 108.66 (95%UI: 85.47–145.29) for males, still higher than 45.02 (95%UI: 36.46–55.79) for females. The peak number of new cases and DALYs cases of TB in China appeared in the 65–69 years age group, which were 80,300 cases (95%UI: 58,100 to 103,300) and 178,200 cases (95%UI: 138,800 to 226,000), respectively. The peak number of deaths occurred in the 70–74 years age group, with 5,300 cases (95%UI: 4,100 to 7,100) (Figure 2 and Supplementary Tables S1–S3).

### 3.2 Trend analysis of TB burden in China from 1990 to 2021

#### 3.2.1 Subgroup trend analysis by age group and gender

**Figure 3:**
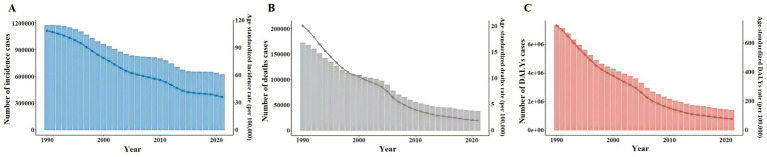
Number and ASR of TB incidence, mortality, and DALYs in China from 1990 to 2021. **(A)** ASIR, Number of incidence cases. **(B)** ASDR, Number of deaths cases. **(C)** ASR of DALYs, Number of DALYs incidence cases.

**Figure 4:**
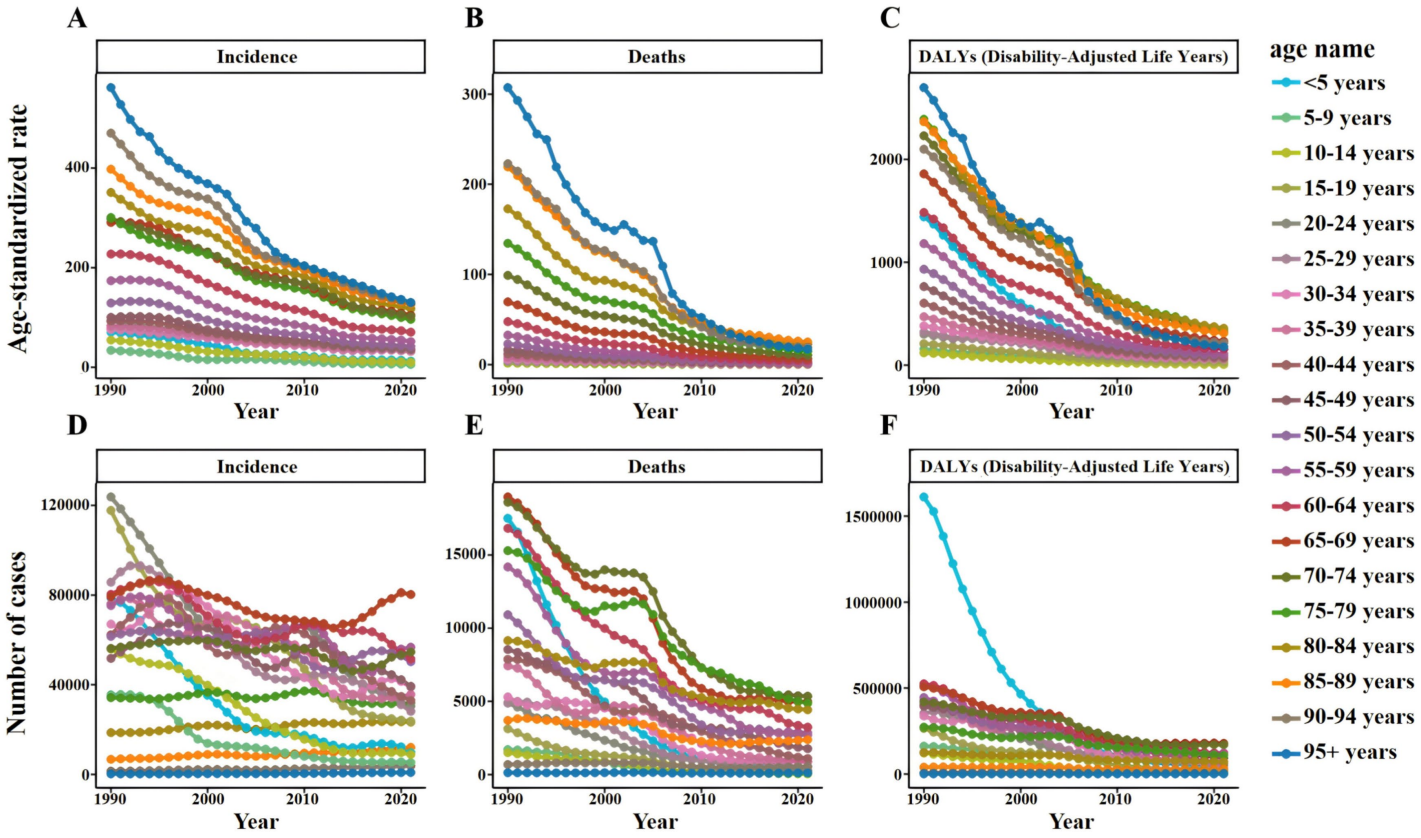
Number and ASR of TB incidence, mortality, and DALYs in China from 1990 to 2021, categorized by age groups **(A)** ASIR. **(B)** ASDR. **(C)** ASR of DALYs. **(D)** Number of incidence cases. **(E)** Number of deaths cases. **(F)** Number of DALYs incidence cases.

Overall, the ASIR of TB decreased from 109.01 per 100,000 in 1990 to 36.28 per 100,000 in 2021. During the same period, ASDR decreased from 20.09 per 100,000 in 1990 to 1.91 per 100,000 in 2021, and ASR of DALYs decreased from 719.42 per 100,000 in 1990 to 76.22 per 100,000 in 2021 (Figure 3). ASIR, ASDR and ASR of DALYs of TB in different age groups showed a downward trend with time (Figure 4).

**Figure 5:**
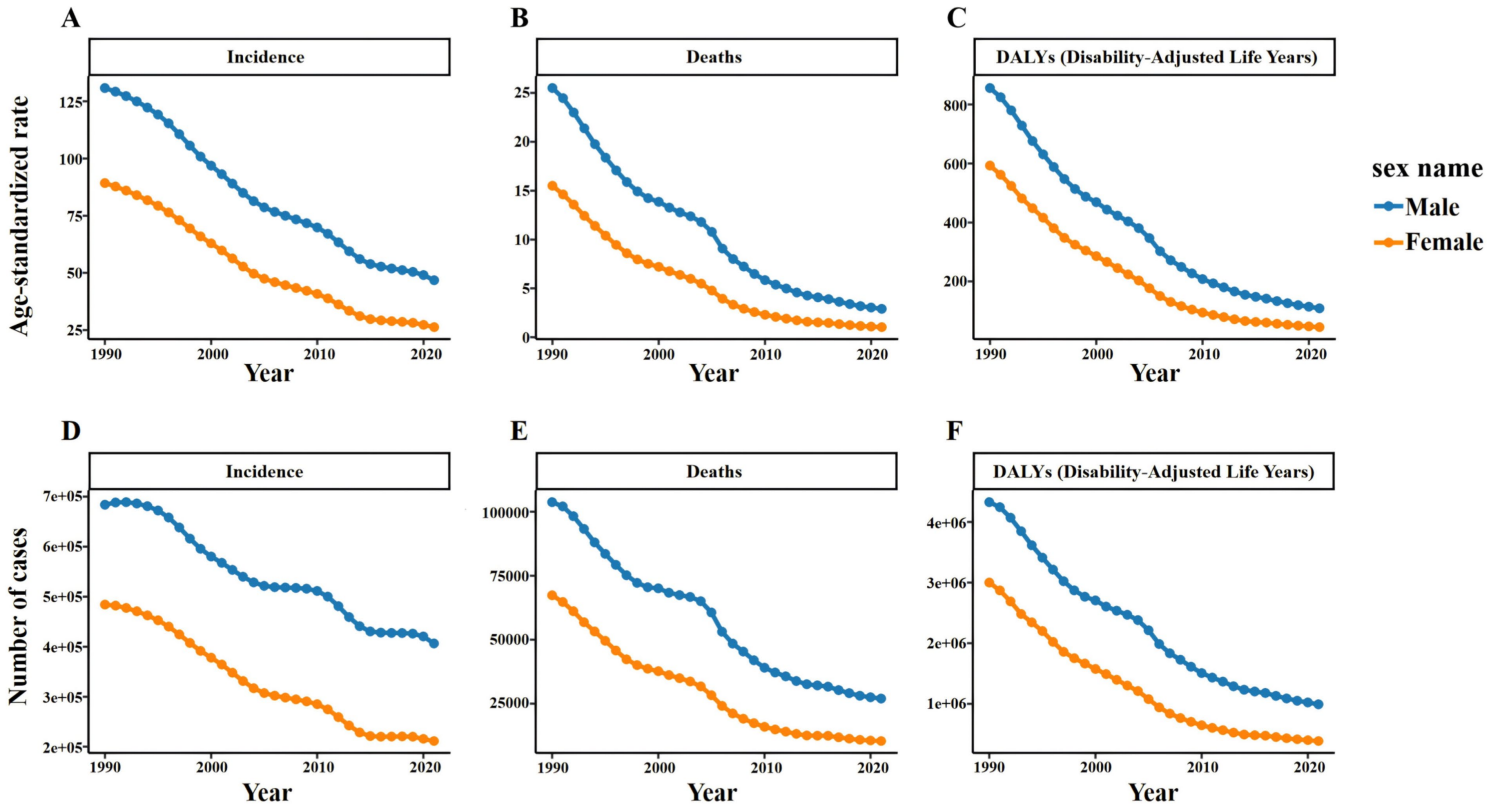
Number and ASR of TB incidence, mortality, and DALYs in China from 1990 to 2021, categorized by gender **(A)** ASIR. **(B)** ASDR. **(C)** ASR of DALYs. **(D)** Number of incidence cases. **(E)** Number of deaths cases. **(F)** Number of DALYs incidence cases.

Specifically, the ASIR of TB in men decreased from 130.77 per 100,000 people in 1990 to 46.75 per 100,000 people in 2021, while that in women decreased from 89.27 per 100,000 people to 26.27 per 100,000 people. During the same period, the ASDR of TB in males decreased from 25.49 per 100,000 to 2.9 per 100,000, and that in females decreased from 15.5 per 100,000 to 1.03 per 100,000. The ASR of DALYs of TB in males decreased from 855.49 per 100,000 to 108.66 per 100,000, and in females decreased from 592.86 per 100,000 to 45.02 per 100,000 (Supplementary Table S5). Similar trends can also be observed in Number by gender, but these trends fluctuate slightly (Figure 5).

#### 3.2.2 Joinpoint regression analysis

**Figure 6:**
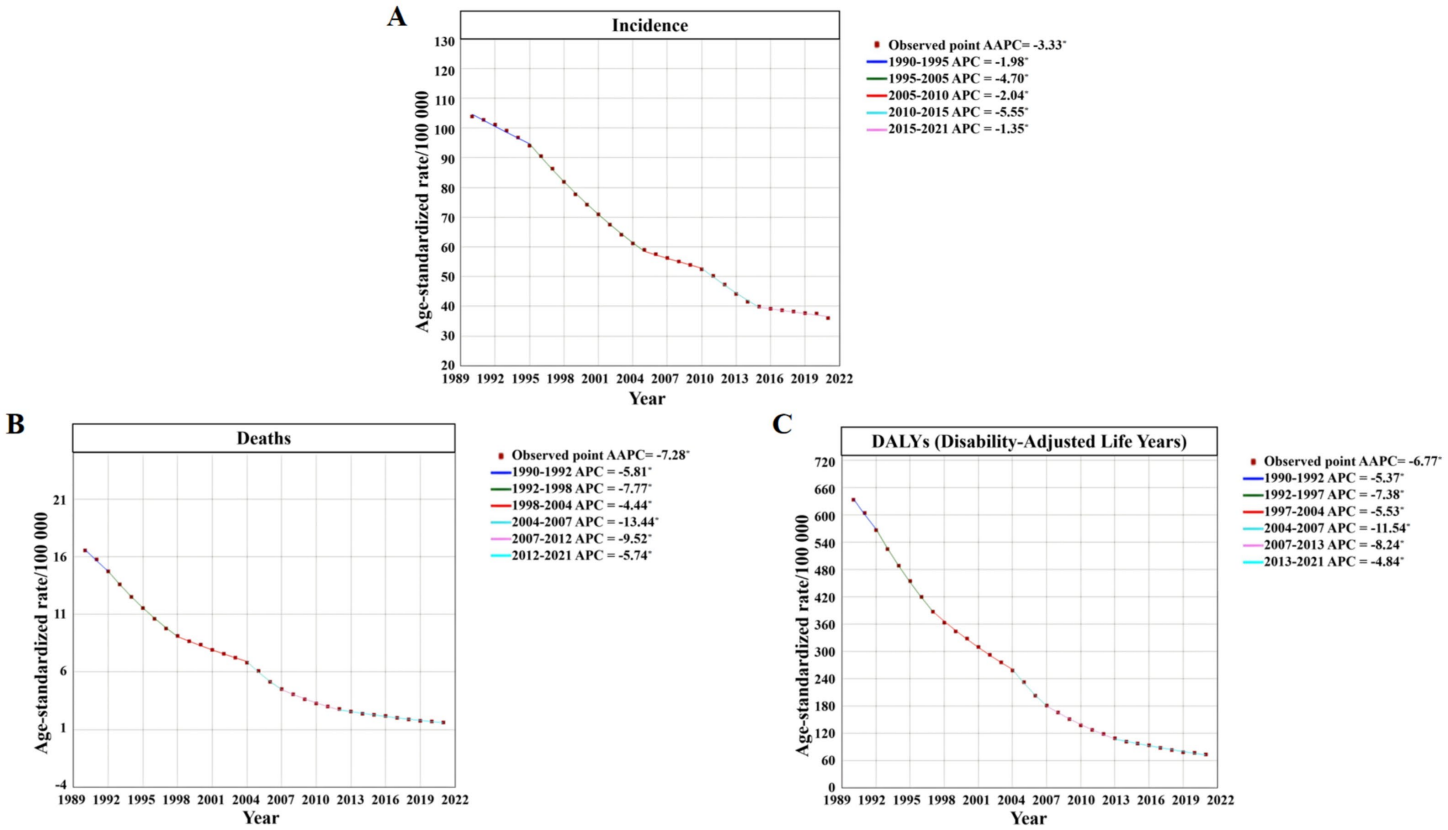
Joinpoint regression analysis of the ASR trends for TB incidence, mortality, and DALYs in China from 1992 to 2021 **(A)** ASIR. **(B)** ASDR. **(C)** ASR of DALYs.

Figure 6 shows the results of Joinpoint regression analysis of TB burden in China from 1990 to 2021. It can be seen that the overall trend of ASIR, ASDR and ASR of DALYs of TB in China decreased year by year from 1990 to 2021. It is worth noting that ASIR decreased significantly during 2010–2015 (APC = −5.55, 95%*CI*: −6.01 to −5.09), and ASDR and ASR of DALYs decreased significantly during 2004–2007 (APC = −13.44, 95%*CI*: −15.40 to −11.44; APC = −11.54, 95% *CI*: −13.30 to −9.75).

From 1990 to 2021, the AAPC of ASIR of TB in China was −3.33 (95%*CI*: −3.45 to −3.21), the AAPC of ASDR was −7.28 (95%*CI*: −7.55 to −7.01), and the AAPC of ASR of DALYs was −6.77 (95%*CI*: −7.00 to −6.54), all *p* < 0.05, indicating a significant decrease in the overall trend (Supplementary Table S5).

### 3.3 APC analysis of TB burden in China from 1992 to 2021

The differences in the incidence rate, mortality and DALYs rate of TB among the total population, males and females in China from 1992 to 2021 were statistically significant (all *p* < 0.05), suggesting that the changes in the incidence rate, mortality and DALYs rate of TB in China were affected by age, period and cohort factors.

**Figure 7:**
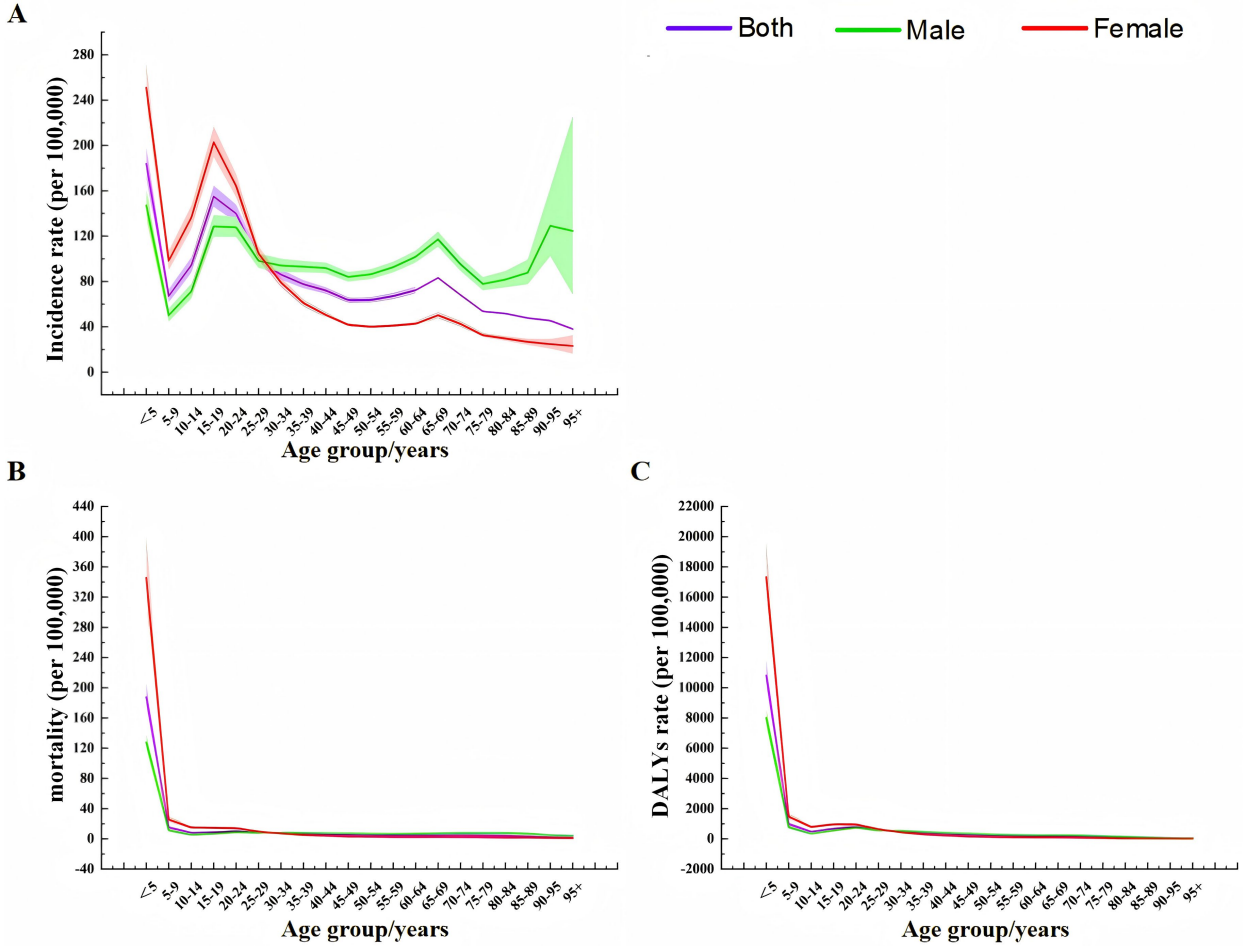
Age effect analysis of TB incidence, mortality, and DALY rates in China from 1990 to 2021 **(A)** Incidence rate. **(B)** Mortality. **(C)** DALYs rate.

The results of age effect analysis (Figure 7) showed that the incidence of TB in the total population, men and women had a similar trend, showing a trend of decreasing first, then increasing and then decreasing. The mortality and DALYs rate also had a similar trend, both showing a trend of rapid decline and then slow fluctuation, and both reached the highest value in the <5 years age group. In general, the age effect of female incidence rate, mortality and DALYs rate was larger than that of male, and the age effect of male was lower than that of female before the age group of 25–29 years, and then male was higher than female (Table 1).

**Table 1:** Age effects of TB incidence, mortality and DALY rate in China from 1990 to 2021.

Factor	Incidence		Deaths		DALYs	
	Rate (per 100,000)	95%CI	Rate (per 100,000)	95%CI	Rate (per 100,000)	95%CI
Age (years)						
<5	183.87	170.01 to 198.86	187.53	170.58 to 206.16	10818.59	9860.13 to 11870.21
5-9	67.26	61.71 to 73.30	15.20	13.65 to 16.92	983.92	886.18 to 1092.44
10-14	94.44	88.01 to 101.35	8.16	7.37 to 9.04	481.95	433.86 to 535.37
15-19	154.95	145.98 to 164.47	9.01	8.31 to 9.77	683.77	629.19 to 743.07
20-24	139.89	132.32 to 147.89	10.32	9.68 to 11.01	796.77	742.80 to 854.67
25-29	99.05	93.92 to 104.47	8.37	7.91 to 8.85	572.32	536.79 to 610.20
30-34	86.11	81.86 to 90.59	7.55	7.18 to 7.93	472.25	445.17 to 500.98
35-39	77.59	74.26 to 81.08	6.47	6.21 to 6.73	379.61	361.56 to 398.57
40-44	72.04	69.06 to 75.16	5.89	5.68 to 6.12	313.05	298.62 to 328.17
45-49	63.57	60.92 to 66.34	5.32	5.13 to 5.51	259.88	247.94 to 272.38
50-54	63.70	61.05 to 66.45	4.76	4.59 to 4.94	209.96	199.87 to 220.57
55-59	67.17	64.33 to 70.14	4.50	4.34 to 4.67	181.75	172.61 to 191.37
60-64	72.35	69.22 to 75.61	4.52	4.35 to 4.69	164.98	156.35 to 174.09
65-69	83.19	79.26 to 87.32	4.73	4.54 to 4.92	160.87	151.89 to 170.39
70-74	68.05	64.48 to 71.83	4.84	4.65 to 5.04	147.98	139.19 to 157.34
75-79	53.59	50.35 to 57.04	4.55	4.36 to 4.75	111.54	104.11 to 119.50
80-84	51.73	48.07 to 55.66	4.06	3.88 to 4.26	77.86	71.66 to 84.60
85-89	47.77	43.23 to 52.80	3.37	3.19 to 3.57	51.98	46.14 to 58.56
90-94	45.48	38.30 to 54.00	2.21	2.02 to 2.43	31.11	24.57 to 39.39
95 plus	38.1	25.81 to 56.23	1.66	1.37 to 2.01	21.92	12.58 to 38.22

**Figure 8:**
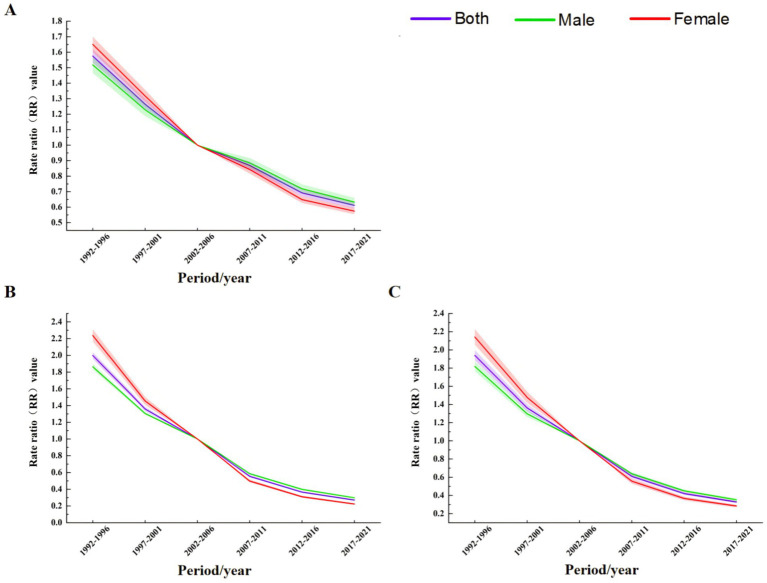
Period effect analysis of TB incidence, mortality, and DALY rates in China from 1990 to 2021 **(A)** Incidence rate. **(B)** Mortality. **(C)** DALYs rate.

The results of period effect analysis (Figure 8) showed that the period effects of TB incidence rate, mortality and DALYs rate risk in the total population, men and women showed a continuous downward trend. The model defaulted to the risk ratio (RR) of TB incidence rate, mortality and DALYs rate in 2002–2006 as the control, that is, RR = 1 in 2002–2006. During 1992–2001, the RR values of TB in the total population, males and females were all greater than 1, and the RR values during 2007–2021 were all less than 1. Before 2002–2006, the RR value of TB in males was lower than that in females, and then the male value was gradually higher than the female value (Table 2).

**Table 2:** The period and cohort effects of TB incidence, mortality and DALY rate in China from 1990 to 2021.

Factor	Incidence		Deaths		DALYs	
	RR	95%CI	RR	95%CI	RR	95%CI
Period						
1992-1996	1.58	1.53 to 1.62	2.00	1.96 to 2.04	1.94	1.88 to 2.00
1997-2001	1.26	1.23 to 1.30	1.36	1.33 to 1.39	1.36	1.32 to 1.40
2002-2006	1	/	1	/	1	/
2007-2011	0.87	0.84 to 0.89	0.56	0.54 to 0.57	0.61	0.59 to 0.63
2012-2016	0.69	0.67 to 0.71	0.37	0.36 to 0.38	0.42	0.41 to 0.44
2017-2021	0.61	0.59 to 0.63	0.27	0.26 to 0.28	0.33	0.31 to 0.34
Cohort						
1897-1901	8.28	2.33 to 29.47	105.42	67.52 to 164.60	69.34	18.14 to 265.15
1902-1906	7.45	4.78 to 11.60	74.52	62.76 to 88.49	49.71	30.53 to 80.93
1907-1911	6.54	5.31 to 8.05	50.34	46.20 to 54.85	34.87	28.18 to 43.15
1912-1916	5.62	4.96 to 6.37	33.31	31.34 to 35.41	23.85	20.99 to 27.09
1917-1921	4.82	4.40 to 5.28	22.93	21.79 to 24.13	16.92	15.42 to 18.56
1922-1926	4.05	3.77 to 4.36	15.95	15.24 to 16.70	12.11	11.22 to 13.06
1927-1931	3.39	3.19 to 3.60	11.37	10.89 to 11.87	9.01	8.43 to 9.64
1932-1936	2.86	2.71 to 3.02	7.95	7.64 to 8.28	6.66	6.26 to 7.08
1937-1941	2.42	2.31 to 2.55	5.52	5.31 to 5.75	4.87	4.59 to 5.15
1942-1946	1.98	1.88 to 2.07	3.58	3.45 to 3.73	3.34	3.16 to 3.53
1947-1951	1.55	1.48 to 1.62	2.25	2.16 to 2.34	2.19	2.08 to 2.31
1952-1956	1.24	1.19 to 1.30	1.46	1.40 to 1.51	1.45	1.38 to 1.52
1957-1961	1	/	1	/	1	/
1962-1966	0.81	0.77 to 0.85	0.65	0.62 to 0.68	0.67	0.64 to 0.71
1967-1971	0.68	0.65 to 0.71	0.45	0.43 to 0.47	0.48	0.46 to 0.51
1972-1976	0.58	0.55to 0.61	0.32	0.30 to 0.33	0.35	0.33 to 0.37
1977-1981	0.50	0.48 to 0.53	0.22	0.21 to 0.23	0.25	0.24 to 0.27
1982-1986	0.43	0.41 to 0.46	0.14	0.13 to 0.15	0.18	0.16 to 0.19
1987-1991	0.36	0.34 to 0.39	0.09	0.08 to 0.09	0.12	0.11 to 0.13
1992-1996	0.30	0.28 to 0.32	0.06	0.06 to 0.07	0.09	0.09 to 0.10
1997-2001	0.25	0.23 to 0.27	0.04	0.03 to 0.04	0.06	0.05 to 0.06
2002-2006	0.18	0.16 to 0.20	0.02	0.02 to 0.02	0.03	0.03 to 0.04
2007-2011	0.13	0.12 to 0.15	0.01	0.01 to 0.01	0.02	0.01 to 0.02
2012-2016	0.10	0.08 to 0.11	0.01	0.00 to 0.01	0.01	0.01 to 0.01
2017-2021	0.09	0.07 to 0.10	0	0.00 to 0.00	0	0.00 to 0.01

**Figure 9:**
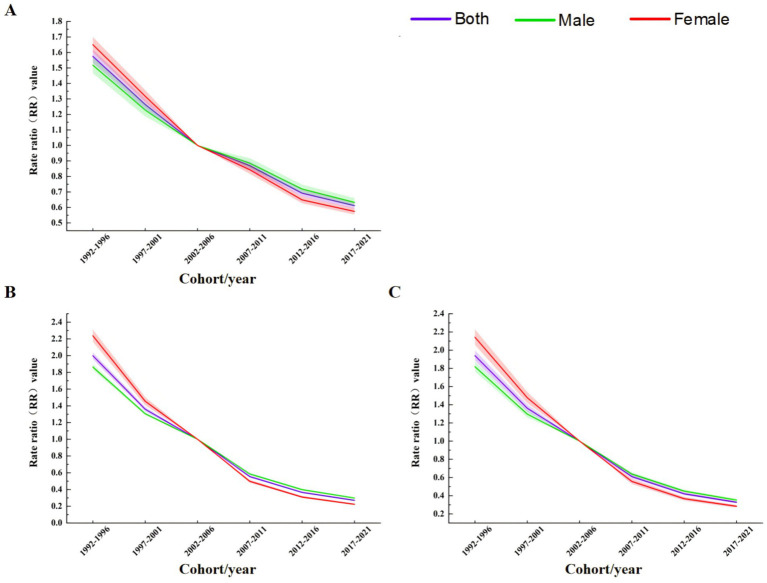
Birth cohort effect analysis of TB incidence, mortality, and DALY rates in China from 1990 to 2021 **(A)** Incidence rate. **(B)** Mortality. **(C)** DALYs rate.

The results of cohort effect analysis showed that (Figure 9), the cohort effects of TB incidence rate, mortality and DALYs rate risk in the total population, men and women showed a continuous downward trend. In the 1897–1902 cohort, the risk of total population, males and females was the highest, and the risk was the lowest in the 2017–2021 cohort (Table 2).

### 3.4 Trend prediction analysis of China’s TB burden from 2022 to 2046

#### 3.4.1 APC model predictions

**Figure 10:**
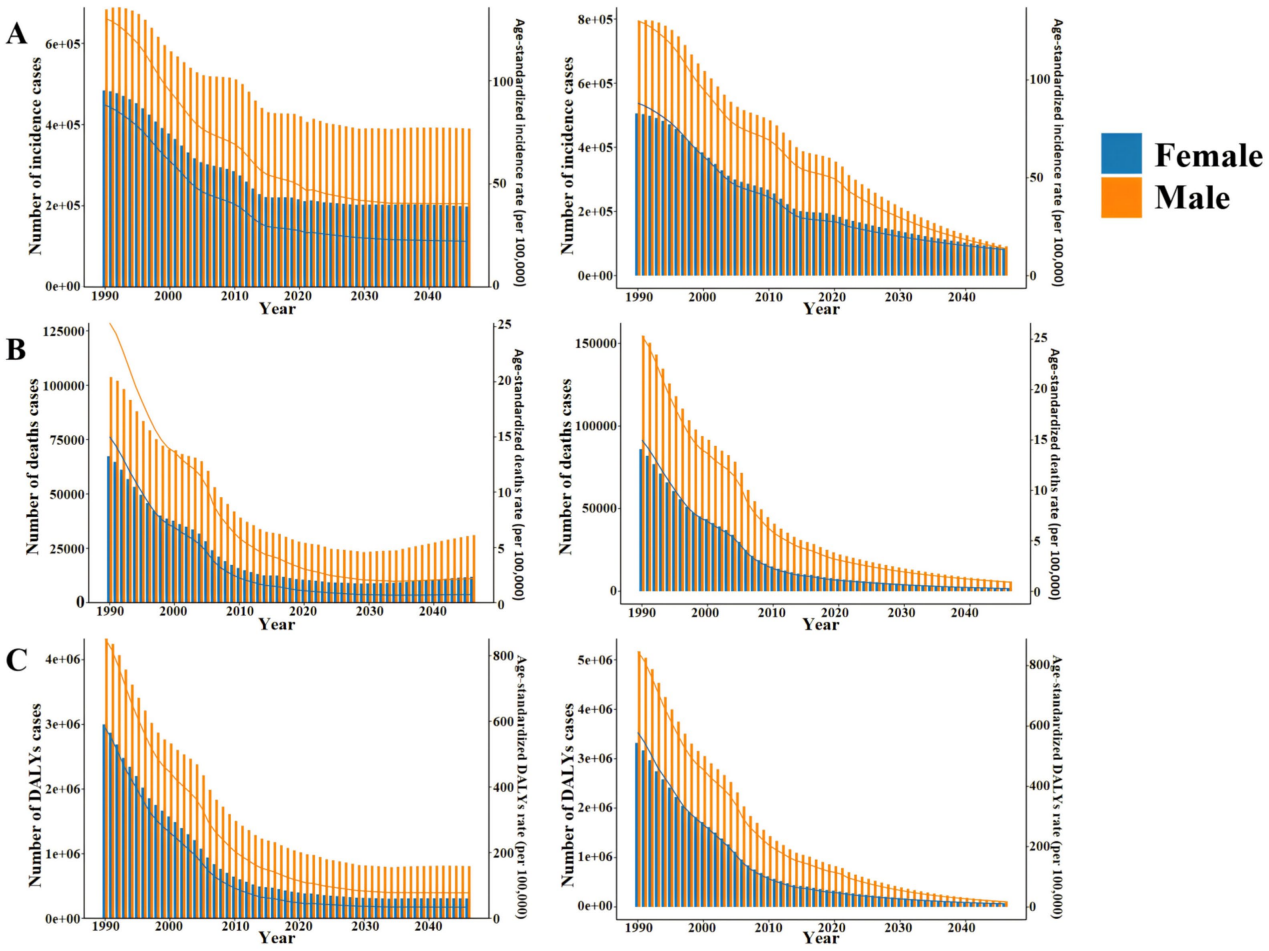
Number and ASR of TB incidence, mortality, and DALYs in China from 2022 to 2046 **(A)** APC model. **(B)** BAPC model.

The prediction results of the APC model (Figure 10A) show that the ASIR, ASDR and ASR of DALYs of TB in Chinese males and females are expected to decrease year by year from 2022 to 2046. The ASIR is expected to be 40.40 per 100,000 and 22.18 per 100,000 in 2046, which is 13.86% and 15.79% lower than that in 2021, respectively. In 2046, ASDR was 2.18 per 100,000 and 0.74 per 100,000, respectively, which was 25.09% and 28.16% lower than that in 2021. In 2046, ASR of DALYs were 77.86 per 100,000 and 33.99 per 100,000, respectively, which were 27.99% and 23.62% lower than those in 2021. During this period, the decline of ASIR and ASDR in males was higher than that in females, while the decline of ASR of DALYs in males was lower than that in females. It is predicted that the number of TB incidence, deaths and DALYs cases in China will continue to decline year by year from 2022 to 2046, reaching 587,900, 42,940 and 1,113,400, respectively, in 2046 (Supplementary Table S6).

#### 3.4.2 BAPC model predictions

The prediction results of the BAPC model (Figure 10B) show that the ASIR, ASDR and ASR of DALYs of TB in Chinese males and females are expected to decrease year by year from 2022 to 2046. The ASIR is expected to be 13.90 per 100,000 and 13.26 per 100,000 in 2046, respectively, which is 70.51% and 50.05% lower than that in 2021. In 2046, ASDR was 0.90 per 100,000 and 0.27 per 100,000, respectively, which were 69.38% and 74.29% lower than those in 2021. In 2046, the ASR of DALYs were 17.38 per 100,000 and 10.44 per 100,000, respectively, which were 84.08% and 76.94% lower than those in 2021. Different from the prediction results of APC model, during this period, the decline of ASIR and ASR of DALYs in males was higher than that in females, while the decline of ASDR in males was lower than that in females. It is predicted that the number of TB incidence, deaths and DALYs cases in China will continue to decline year by year from 2022 to 2046, reaching 177,100, 7,700 and 181,700, respectively, in 2046 (Supplementary Table S7).

## 4 Discussion

Our study found that in 2021, the Number and ASR values of TB incidence, death and DALYs in males were higher than those in females in almost all age groups. This finding is consistent with the 2024 global tuberculosis report provided by WHO (1) which shows that the number of TB incidence, deaths and DALYs in men is higher than that in women. It can be seen that TB has a higher health impact on Chinese men than women, which is similar to the results of Eksombatchai et al. (27). The daily life of men is usually more closely related to smoking, alcohol abuse and long-term high-intensity work stress. Studies have shown that these are highly correlated with the increased risk of TB (28). Hsien-Ho et al. (29) showed that smoking was one of the main risk factors for TB in Taiwan, and the probability of TB in smokers was more than twice that of non-smokers. Long-term high-intensity physical labor, mental stress, not a good rest is likely to lead to reduced immunity, which is more likely to be infected with TB (30). ASIR, ASDR, and ASR of DALYs usually increased with age and peaked in the >60 years age group, reflecting a higher risk of TB burden in the older adult. Studies conducted by Zhang Ting et al. (31) show that the global burden of TB mainly affects middle-aged and older adult groups. The immune and metabolic capacity of the older adult population after the age of 60 is reduced, and most of them are accompanied by other chronic diseases. The symptoms of TB are often mistaken for a cold, which cannot be diagnosed and treated in time, resulting in a high potential risk. Therefore, we should do a good job in health education and publicity for the whole population, and strengthen the screening of TB patients in key populations, especially in the older adult, so as to ensure early detection, timely diagnosis and treatment. It is worth noting that this study provides a more detailed and in-depth analysis of demographic information than other studies (17, 32).

In addition, compared with the traditional regression model, the Joinpoint regression model can not only analyze the overall trend of the data, but also reveal its local trend changes (33), providing more accurate and more detailed information (34, 35). From 1990 to 2021, the three indicators of ASIR, ASDR and ASR of DALYs showed an overall downward trend year by year. ASIR decreased significantly in 2010–2015, ASDR and ASR of DALYs decreased significantly in 2004–2007, which was consistent with the global tuberculosis report in 2024 (1). This may be related to the release and implementation of a series of health policies related to TB prevention and control in China from 2004 to 2015 and the improvement of citizens ‘awareness of self-protection. In particular, the implementation of the “National Tuberculosis Prevention and Control Plan (2011–2015)” (36) issued by the General Office of the State Council of China has steadily promoted the implementation of China’s TB control strategy to effectively control the TB epidemic. In addition, the rapid development of China’s economy and culture during this period and the popularization of BCG vaccine have effectively reduced the TB burden in China to a certain extent.

The APC model study determined the APC effect of TB in China. The results of the age effect showed that from 1990 to 2021, with the increase of age, the incidence of TB in China’s total population, men and women decreased first, then increased and then decreased, while the mortality and DALYs rate decreased rapidly first and then decreased slowly, and both reached the highest value in the <5 years age group. The risk of incidence in the 15–24 age group showed a small peak. This may be due to the fact that this age group is mainly a student group. Schools usually screen for TB when freshmen are enrolled in physical examination. Students are long-term gathered in the classroom and poorly ventilated, which may lead to an increase in the risk of TB transmission. In addition, after the age of 60, the overall population, male and female incidence rates showed a rapid growth trend, similar to previous research results (37, 38). The reason may be related to the increase of population aging and the decrease of immune function of the older adult themselves. The results of period effect showed that the incidence rate, mortality and DALYs rate risk of TB in China showed a downward trend. This shows that with the continuous improvement of China’s medical and health technology, people will pay more attention to improving self-prevention and health awareness. The improvement of China’s economic conditions in recent years may have a certain effect on TB incidence rate, mortality and DALYs rate risk reduction. This is similar to the conclusion of Wang et al. (39) that the level of GDP per capita has a certain impact on the TB burden. The results of cohort effect showed that the risk of TB incidence rate, mortality and DALYs rate in China decreased significantly. That is, at the same age, the later the birth, the lower the risk of TB incidence rate, mortality and DALYs rate. This may be related to the following reasons: in the mid-1970s, China included BCG vaccine for TB prevention in children’s immunization programs, and the neonatal vaccination rate reached 90% in 2000; after the SARS epidemic was controlled in 2003, the Chinese government strengthened the management of the public health system, revised the laws and regulations related to infectious diseases, and increased the Internet disease reporting system, which provided better protection for the prevention and treatment of TB. Compared with people born in the era of social unrest, people born in a socially stable environment have a higher level of health (40). However, these results are only based on the data studied in the GBD 2021 database, and the actual situation may be different, which requires targeted data collection and analysis in the future.

The trend prediction based on APC model shows that the ASIR, ASDR and ASR of DALYs of TB in China will decrease year by year and gradually stabilize. The APC model predicts that the number of deaths in TB will show a trend of decreasing first and then increasing, predicting that there will be about 42,900 deaths by 2046. This increase is mainly due to the aging of the population and the increase in life expectancy (41). The BAPC model provides a more positive and optimistic forecast prospect, predicting that there will be 177,100 new cases, 7,700 deaths and 181,700 DALYs cases by 2046, which may be due to the continuous progress of TB treatment methods and the improvement of China’s health care system. With the further deepening of population aging, China’s relevant health departments may promote more strategies to prevent TB, thus effectively reducing the incidence of TB in the older adult. With the further deepening of population aging, it is suggested that the relevant health departments in China should promote more strategies to prevent TB, so as to effectively reduce the incidence of PB in the older adult. In particular, we should pay attention to strengthening the promotion and implementation of TB in underdeveloped areas, high-incidence areas of tuberculosis and rural towns in central and western China.

This study included 32 years of TB incidence rate, mortality and DALYs rate data in China from 1990 to 2021 in GBD 2021 database. The main advantages include a large database covering the entire population of China and a wide time span, comparing the TB burden of different age groups, and considering the differences between genders, which is conducive to mastering the epidemic law of the disease and providing a theoretical reference for better TB prevention and control in China. There are still some limitations in the research. First of all, the GBD 2021 database does not include relevant data information stratified by different provinces, cities and rural areas, which limits the ability to explore more detailed information affecting TB epidemic trends in China. Secondly, the ASIR, ASDR and ASR of DALYs of TB are calculated using the GBD standard population rather than the Chinese population. Although it is conducive to horizontal comparison with other countries, due to the large proportion of the older adult population in China, the specific values may be lower than the actual. Finally, this study is based on historical data and current trends, without fully considering potential changes in the direction of new medical technologies and policies that may affect future trends. Therefore, future research must consider these potential trend factors.

In conclusion, TB, as one of the oldest infectious diseases, has always been the focus of prevention and treatment of infectious diseases in China. ASIR, ASDR and ASR of DALYs all show a downward trend year by year, and the corresponding ASR of males is higher than that of females. The age effect of TB risk in China increased first and then decreased with age. The age effect of death and DALYs risk decreased rapidly and then slowly with age. The period effect and cohort effect decreased with time. The prediction of APC and BAPC models shows that the ASR of TB in China will decrease year by year. In addition, men and the older adult are still at high risk of TB burden. It is recommended to strengthen the screening of TB patients in key populations, especially in the older adult, in combination with the promotion and application of new technologies.

## Data Availability

The original contributions presented in the study are included in the article/Supplementary material, further inquiries can be directed to the corresponding author.

## Footnotes

^1^ https://ghdx.healthdata.org/gbd-2021

^2^ https://analysistools.nci.nih.gov/apc/
