# Supplementary material for: Age-period-cohort analysis and prediction of tuberculosis trends in China—based on the Global Burden of Disease 2021 data
Source: Front Public Health. 2025 Feb 14;13:1512514. doi: 10.3389/fpubh.2025.1512514 (PMC11868063; doi:10.3389/fpubh.2025.1512514)
Supplement: Supplementary file 1 [file Data_Sheet_1.pdf]

Age-period-cohort analysis and prediction of tuberculosis trends in China—based on the Global Burden of Disease 2021 data

Supplementary Materials

Table S1 Number and ASR of TB incidence in China in 2021 1

Table S2 Number and ASR of TB deaths in China in 2021 2

Table S3 Number and ASR of TB DALYs in China in 2021 3

Table S4 Number and ASR of TB incidence, mortality, and DALYs in China from 1990 to 2021 4

Table S5 Average Annual Percentage Change of the ASR for TB incidence, mortality, and DALYs in China from 1990 to 2021, categorized by gender 5

Table S6 Number and ASR prediction of TB incidence, mortality and DALY in China from 2022 to 2046 based on APC model 6

Table S7 Number and ASR prediction of TB incidence, mortality and DALY in China from 2022 to 2046 based on BAPC model 7

Table S1 Number and ASR of TB incidence in China in 2021

	**Incidence**				
	**Num_1990**	**ASR_1990 (per 100,000)**	**Num_2021**	**ASR_2021 (per 100,000)**	**EAPC_CI**
**China**	1167808 (1001441 to 1359621)	109.01 (94.81 to 124.61)	617726 (549548 to 688348)	36.28 (32.63 to 40.47)	-3.78 (-3.89 to -3.67)
**Gender**					
Male	683686 (593332 to 789780)	130.77 (113.94 to 149.71)	406433 (360732 to 452642)	46.75 (41.84 to 51.91)	-4.25 (-4.39 to -4.11)
Female	484122 (407126 to 574804)	89.27 (76.56 to 103.22)	211293 (186374 to 236357)	26.27 (23.19 to 29.49)	-3.49 (-3.59 to -3.4)
**age_name**					

<5 years	78692 (58809 to 101095)	70.38 (52.6 to 90.42)	9714 (7664 to 12247)	12.51 (9.87 to 15.77)	-5.85 (-6.14 to -5.55)
05 to 9 years	35421 (22103 to 52487)	33.97 (21.2 to 50.33)	5296 (3480 to 7837)	5.53 (3.63 to 8.18)	-5.7 (-6.07 to -5.34)
10 to 14 years	55868 (34628 to 81961)	54.62 (33.85 to 80.12)	8896 (5488 to 13413)	10.32 (6.37 to 15.56)	-5.46 (-5.65 to -5.27)
15 to 19 years	117709 (68706 to 173944)	92.93 (54.24 to 137.33)	23710 (15415 to 33892)	31.75 (20.64 to 45.39)	-3.5 (-3.61 to -3.38)
20 to 24 years	123723 (71464 to 191194)	93.73 (54.14 to 144.84)	30396 (18914 to 43091)	41.54 (25.85 to 58.89)	-2.67 (-2.83 to -2.5)
25 to 29 years	85724 (49621 to 131307)	78.01 (45.16 to 119.49)	28082 (17827 to 42149)	32.47 (20.61 to 48.74)	-3.06 (-3.21 to -2.91)
30 to 34 years	67050 (42130 to 98277)	75.98 (47.74 to 111.37)	38932 (25614 to 54533)	32.13 (21.14 to 45.01)	-2.99 (-3.1 to -2.87)
35 to 39 years	76040 (44520 to 111816)	83.25 (48.74 to 122.42)	35639 (22770 to 48927)	33.63 (21.49 to 46.17)	-3.16 (-3.29 to -3.03)
40 to 44 years	62192 (37752 to 94346)	92.69 (56.27 to 140.62)	32935 (21842 to 45862)	35.98 (23.86 to 50.1)	-3.39 (-3.52 to -3.26)
45 to 49 years	51690 (31569 to 78447)	100.14 (61.16 to 151.97)	39238 (27662 to 53717)	35.57 (25.07 to 48.69)	-3.78 (-3.93 to -3.62)
50 to 54 years	61496 (38074 to 90584)	128.89 (79.8 to 189.86)	50214 (33922 to 68264)	41.55 (28.07 to 56.48)	-4.09 (-4.25 to -3.94)
55 to 59 years	75331 (46849 to 110454)	173.7 (108.02 to 254.68)	56675 (39276 to 75389)	51.55 (35.72 to 68.57)	-4.34 (-4.51 to -4.17)
60 to 64 years	80303 (48559 to 117812)	227.25 (137.41 to 333.39)	51400 (34719 to 71457)	70.41 (47.56 to 97.88)	-4.17 (-4.32 to -4.03)
65 to 69 years	79293 (49677 to 109063)	290.64 (182.09 to 399.76)	80256 (58138 to 103330)	104.63 (75.8 to 134.71)	-3.57 (-3.72 to -3.43)
70 to 74 years	56117 (37575 to 76931)	298.21 (199.68 to 408.82)	54571 (38629 to 72165)	102.39 (72.48 to 135.4)	-3.53 (-3.68 to -3.39)
75 to 79 years	34195 (22255 to 47485)	300.47 (195.55 to 417.24)	31827 (21941 to 42706)	96.1 (66.25 to 128.95)	-3.65 (-3.77 to -3.53)
80 to 84 years	18589 (14028 to 24138)	350.92 (264.83 to 455.68)	23278 (17819 to 28728)	117.61 (90.03 to 145.15)	-3.57 (-3.7 to -3.44)
85 to 89 years	6707 (5134 to 8465)	397.6 (304.33 to 501.82)	12047 (9570 to 14782)	126.47 (100.47 to 155.18)	-3.75 (-3.88 to -3.61)
90 to 94 years	1442 (1014 to 1870)	469.91 (330.55 to 609.38)	3788 (2870 to 4875)	129.18 (97.9 to 166.26)	-4.15 (-4.28 to -4)
95+ years	228 (136 to 315)	561.88 (336.26 to 777.58)	832 (548 to 1179)	130.14 (85.81 to 184.48)	-4.7 (-4.84 to -4.55)

Table S2 Number and ASR of TB deaths in China in 2021

	**Deaths**				
	**Num_1990**	**ASR_1990 (per 100,000)**	**Num_2021**	**ASR_2021 (per 100,000)**	**EAPC_CI**
**China**	171091 (141634 to 204908)	20.09 (16.68 to 23.84)	37332 (29309 to 49368)	1.91 (1.51 to 2.51)	-7.79 (-8.06 to -7.51)
**Gender**					
Male	103742 (77568 to 132622)	25.49 (18.96 to 32.55)	26981 (19576 to 38551)	2.9 (2.13 to 4.12)	-7.19 (-7.46 to -6.91)
Female	67349 (56026 to 79053)	15.5 (12.96 to 18.11)	10351 (7976 to 13860)	1.03 (0.79 to 1.37)	-8.99 (-9.31 to -8.67)
**age_name**					
<5 years	17501 (14612 to 20663)	15.65 (13.07 to 18.48)	210 (165 to 268)	0.27 (0.21 to 0.34)	-12.57 (-13 to -12.14)
05 to 9 years	1702 (1414 to 1999)	1.63 (1.36 to 1.92)	46 (38 to 58)	0.05 (0.04 to 0.06)	-11.44 (-12.04 to -10.84)
10 to 14 years	1496 (1254 to 1736)	1.46 (1.23 to 1.7)	55 (46 to 67)	0.06 (0.05 to 0.08)	-9.79 (-10.14 to -9.44)
15 to 19 years	3123 (2605 to 3693)	2.47 (2.06 to 2.92)	134 (108 to 167)	0.18 (0.14 to 0.22)	-8.53 (-8.75 to -8.3)
20 to 24 years	4874 (3965 to 5900)	3.69 (3 to 4.47)	301 (238 to 396)	0.41 (0.32 to 0.54)	-7.72 (-8.22 to -7.22)
25 to 29 years	4886 (3984 to 5855)	4.45 (3.63 to 5.33)	512 (397 to 679)	0.59 (0.46 to 0.78)	-7.37 (-7.86 to -6.88)
30 to 34 years	5297 (4308 to 6247)	6 (4.88 to 7.08)	891 (683 to 1200)	0.74 (0.56 to 0.99)	-7.33 (-7.67 to -7)
35 to 39 years	7417 (6115 to 8990)	8.12 (6.7 to 9.84)	868 (670 to 1169)	0.82 (0.63 to 1.1)	-7.17 (-7.38 to -6.97)
40 to 44 years	7864 (6298 to 9539)	11.72 (9.39 to 14.22)	1096 (832 to 1495)	1.2 (0.91 to 1.63)	-7.37 (-7.57 to -7.17)
45 to 49 years	8531 (6773 to 10397)	16.53 (13.12 to 20.14)	1767 (1281 to 2370)	1.6 (1.16 to 2.15)	-7.72 (-7.97 to -7.48)
50 to 54 years	10919 (8586 to 13400)	22.89 (18 to 28.09)	2704 (1976 to 3637)	2.24 (1.63 to 3.01)	-7.73 (-7.92 to -7.54)

55 to 59 years	14180 (11266 to 17358)	32.7 (25.98 to 40.02)	2842 (2125 to 3814)	2.58 (1.93 to 3.47)	-8.05 (-8.28 to -7.83)
60 to 64 years	16814 (13381 to 20389)	47.58 (37.87 to 57.7)	3232 (2435 to 4418)	4.43 (3.34 to 6.05)	-8.1 (-8.45 to -7.75)
65 to 69 years	18956 (15402 to 23359)	69.48 (56.45 to 85.62)	4981 (3775 to 6655)	6.49 (4.92 to 8.68)	-7.83 (-8.18 to -7.48)
70 to 74 years	18595 (15144 to 22505)	98.82 (80.48 to 119.59)	5344 (4136 to 7070)	10.03 (7.76 to 13.27)	-7.38 (-7.69 to -7.07)
75 to 79 years	15290 (12546 to 18236)	134.35 (110.24 to 160.24)	4874 (3809 to 6472)	14.72 (11.5 to 19.54)	-7.03 (-7.29 to -6.76)
80 to 84 years	9141 (7536 to 10977)	172.57 (142.26 to 207.22)	4417 (3402 to 5789)	22.32 (17.19 to 29.25)	-6.7 (-6.95 to -6.45)
85 to 89 years	3696 (3059 to 4367)	219.11 (181.34 to 258.87)	2385 (1918 to 3004)	25.03 (20.14 to 31.54)	-7.42 (-7.76 to -7.09)
90 to 94 years	683 (561 to 802)	222.59 (182.82 to 261.46)	564 (442 to 691)	19.24 (15.06 to 23.58)	-8.71 (-9.23 to -8.19)
95+ years	124 (98 to 148)	307.29 (242 to 365.01)	109 (78 to 139)	17.02 (12.14 to 21.69)	-9.61 (-10.22 to -9.01)

Table S3 Number and ASR of TB DALYs in China in 2021

	**Disability-Adjusted Life Years（DALYs）**				
	**Num_1990**	**ASR_1990 (per 100,000)**	**Num_2021**	**ASR_2021 (per 100,000)**	**EAPC_CI**
**China**	7325730 (6221452 to 8528411)	719.42 (610.63 to 837.38)	1375510 (1120820 to 1723072)	76.22 (62.59 to 94.45)	-7.49 (-7.7 to -7.27)
**Gender**					
Male	4325971 (3342350 to 5378669)	855.49 (660.79 to 1064.16)	991182 (767265 to 1335566)	108.66 (85.47 to 145.29)	-6.84 (-7.04 to -6.65)
Female	2999759 (2585588 to 3434183)	592.86 (510.12 to 677.66)	384328 (308366 to 483222)	45.02 (36.46 to 55.79)	-8.66 (-8.95 to -8.38)
**age_name**					
<5 years	1611064 (1353446 to 1902099)	1440.95 (1210.53 to 1701.25)	27087 (21989 to 33570)	34.88 (28.31 to 43.22)	-11.77 (-12.1 to -11.44)
05 to 9 years	161766 (135597 to 189616)	155.13 (130.03 to 181.84)	7482 (5699 to 9827)	7.81 (5.95 to 10.26)	-9.87 (-10.3 to -9.44)
10 to 14 years	121631 (102114 to 141549)	118.91 (99.83 to 138.38)	5112 (4252 to 6063)	5.93 (4.93 to 7.03)	-9.45 (-9.76 to -9.14)
15 to 19 years	265089 (222770 to 313517)	209.28 (175.87 to 247.52)	21595 (16091 to 28718)	28.92 (21.55 to 38.46)	-6.66 (-6.9 to -6.42)
20 to 24 years	395698 (322791 to 469553)	299.77 (244.54 to 355.72)	46768 (34017 to 64005)	63.91 (46.49 to 87.47)	-5.66 (-6.1 to -5.22)
25 to 29 years	352652 (288742 to 414344)	320.91 (262.76 to 377.06)	51798 (40152 to 67639)	59.89 (46.43 to 78.21)	-6.12 (-6.49 to -5.75)
30 to 34 years	337230 (275658 to 392759)	382.15 (312.38 to 445.08)	70816 (55807 to 92028)	58.45 (46.06 to 75.96)	-6.55 (-6.82 to -6.27)
35 to 39 years	429818 (353234 to 519044)	470.57 (386.73 to 568.26)	63067 (48222 to 80542)	59.52 (45.51 to 76.01)	-6.53 (-6.69 to -6.38)
40 to 44 years	403928 (328254 to 479508)	602.03 (489.24 to 714.68)	66113 (51190 to 86117)	72.23 (55.93 to 94.08)	-6.9 (-7.08 to -6.71)
45 to 49 years	394076 (320131 to 477306)	763.43 (620.18 to 924.67)	93559 (71160 to 121105)	84.81 (64.5 to 109.77)	-7.3 (-7.51 to -7.09)
50 to 54 years	444674 (360121 to 548764)	932.02 (754.8 to 1150.19)	122669 (93010 to 159193)	101.5 (76.96 to 131.72)	-7.38 (-7.55 to -7.22)
55 to 59 years	512283 (415985 to 612934)	1181.22 (959.17 to 1413.3)	117618 (89091 to 153773)	106.98 (81.03 to 139.87)	-7.67 (-7.86 to -7.48)
60 to 64 years	524232 (426531 to 631629)	1483.5 (1207.02 to 1787.42)	114720 (89112 to 150540)	157.14 (122.06 to 206.21)	-7.62 (-7.91 to -7.32)
65 to 69 years	507033 (418432 to 616202)	1858.5 (1533.74 to 2258.65)	178241 (138826 to 225990)	232.38 (180.99 to 294.63)	-6.84 (-7.1 to -6.58)
70 to 74 years	419416 (349200 to 496995)	2228.85 (1855.7 to 2641.11)	170759 (132535 to 212560)	320.39 (248.67 to 398.83)	-6.23 (-6.43 to -6.02)
75 to 79 years	272096 (227551 to 320747)	2390.86 (1999.44 to 2818.34)	110032 (88115 to 138475)	332.23 (266.06 to 418.11)	-6.23 (-6.43 to -6.03)
80 to 84 years	125575 (105259 to 149256)	2370.63 (1987.1 to 2817.68)	70861 (57775 to 89499)	358.03 (291.91 to 452.2)	-6.19 (-6.4 to -5.98)
85 to 89 years	39937 (33565 to 47089)	2367.52 (1989.82 to 2791.53)	29692 (24493 to 36760)	311.7 (257.12 to 385.9)	-6.94 (-7.24 to -6.63)
90 to 94 years	6438 (5374 to 7527)	2098.36 (1751.37 to 2453.35)	6381 (5210 to 7773)	217.62 (177.69 to 265.1)	-8.05 (-8.5 to -7.59)
95+ years	1093 (881 to 1271)	2699.36 (2174.55 to 3139.7)	1141 (872 to 1400)	178.55 (136.48 to 219.03)	-9.09 (-9.63 to -8.55)

Table S4 Number and ASR of TB incidence, mortality, and DALYs in China from 1990 to 2021

Year	Incidence						Deaths						Disability-Adjusted Life Years（DALYs）					
	ASR (per 100000)			Number			ASR (per 100000)			Number			ASR (per 100000)			Number		
	Both	Male	Female	Both	Male	Female	Both	Male	Female	Both	Male	Female	Both	Male	Female	Both	Male	Female
1990	109.0141776	130.7739329	89.27437549	1167807.741	683685.6254	484122.1157	20.09498577	25.49466476	15.50355191	171090.8226	103742.0075	67348.81505	719.422413	855.4918334	592.8627458	7325730.059	4325971.167	2999758.892
1991	107.5435845	129.1949003	87.75648541	1170243.368	688149.1781	482094.1894	19.15481012	24.45920354	14.61653177	166800.9446	102076.1493	64724.79528	688.7907184	824.451164	561.8218336	7113497.496	4243443.613	2870053.884
1992	105.7590244	127.2576	85.99477448	1166271.198	688822.7051	477448.4932	17.93556531	22.99247544	13.57838052	159363.5734	98251.68728	61111.88614	648.0471552	779.7387539	524.0736354	6758097.427	4068975.888	2689121.54
1993	103.6438083	124.9369411	83.98249159	1156881.874	686129.0864	470752.7876	16.58032294	21.37473214	12.43187302	150079.0613	93266.73156	56812.32975	601.43901	728.1853181	481.6120464	6327201.993	3846067.04	2481134.954
1994	101.247595	122.2525243	81.77880168	1143310.143	680753.8377	462556.305	15.2838775	19.75465892	11.40000448	141217.8803	88009.63721	53208.24313	559.018564	676.0446771	448.4732177	5958463.834	3615180.245	2343283.589
1995	98.53858171	119.2023289	79.35410061	1124920.413	672160.9867	452759.4262	14.11532734	18.37619155	10.40293049	133089.3081	83531.23673	49558.07135	520.5097035	630.9570566	416.2187607	5610557.695	3410814.505	2199743.19
1996	95.17131708	115.3524092	76.45238361	1098226.059	658107.285	440118.7738	13.0049283	17.07160594	9.44814367	125004.2192	79244.17262	45760.04659	481.5917887	588.0760846	380.7239622	5235191.475	3213586.498	2021604.977
1997	91.10739904	110.6380246	73.04541716	1062641.51	638214.4713	424427.0382	11.98753667	15.88025239	8.582863172	117506.5403	75195.67789	42310.86244	445.0046971	547.324533	347.9858992	4879187.643	3022061.88	1857125.763
1998	86.79842726	105.624996	69.45291065	1023526.929	615993.681	407533.2483	11.20090593	14.92296063	7.950678117	112258.4872	72198.19899	40060.28825	416.5759335	513.5713892	324.7344665	4623137.686	2871209.545	1751928.141
1999	82.66568404	100.8531552	65.97077784	987233.5622	595674.366	391559.1962	10.64326847	14.2380741	7.505482003	109122.2273	70479.72605	38642.50129	393.7433543	487.4946417	304.8909857	4430326.5	2766208.256	1664118.244
2000	79.15048119	96.87290082	62.92430696	958173.2172	580156.2187	378016.9985	10.30474135	13.86030251	7.194990593	107745.5397	70064.4879	37681.05179	374.9563484	468.9746761	285.5249723	4279637.927	2704835.268	1574802.66
2001	75.73267531	93.13926599	59.80499481	931905.8981	567557.4771	364348.4211	9.774635533	13.2687708	6.74663574	104492.6818	68341.71649	36150.96533	352.398081	443.7695659	265.8401957	4093363.79	2603523.392	1489840.398
2002	71.93929436	89.03628622	56.25847608	901616.1809	553554.9566	348061.2244	9.339913579	12.78021958	6.366906244	102358.8742	67414.61849	34944.25566	331.8418025	423.332329	244.9729519	3930918.339	2534905.551	1396012.788
2003	68.17687488	84.98267026	52.71512513	871098.5196	539730.0527	331368.4669	8.927721836	12.37850355	5.972682225	100349.7877	66687.44925	33662.33848	311.2955113	403.7200691	223.5651396	3768771.426	2468065.377	1300706.05
2004	64.86617225	81.37832634	49.62370651	845482.9924	528521.6672	316961.3252	8.381089317	11.7956012	5.477593128	96767.11691	65024.09298	31743.02393	289.5319004	380.8498575	202.9545363	3590206.734	2380318.224	1209888.511
2005	62.43920742	78.66391769	47.42401172	828801.235	521469.5578	307331.6772	7.529847462	10.78437037	4.771212178	88909.10729	60620.19632	28288.91097	259.6432917	347.1665015	176.6879196	3289646.793	2212701.326	1076945.467
2006	60.7127841	76.68574171	45.91221605	821099.8652	518968.0485	302131.8166	6.300329907	9.066084457	3.932198058	77228.44449	53099.13129	24129.3132	224.5489253	302.4100869	150.3158633	2927072.76	1986352.491	940720.2691
2007	59.2305137	74.99692043	44.59884151	816430.3817	518153.4634	298276.9182	5.478760442	7.991407565	3.325172942	69637.83336	48468.26965	21169.5637	199.2252746	271.4300898	130.3076179	2671389.379	1833109.236	838280.1424
200	57.84430	73.39634	43.37829	812081.4	517370.8	294710.5	4.909077	7.223904	2.910202	64446.59	45344.73	19101.86	181.1398	249.0162	116.1795	2490723.	1726235.	764488.2

8	05	223	252	511	709	801	402	333	099	761	375	386	751	698	086	478	214	64
2009	56.41640822	71.73805006	42.13787044	806591.7509	515840.722	290751.0289	4.379200664	6.468668678	2.56614363	59304.74932	41937.41877	17367.33055	164.5038837	227.1102679	104.5206088	2313991.979	1611569.757	702422.2221
2010	54.82744669	69.87544182	40.78015046	796441.4563	511360.3003	285081.156	3.946765144	5.834692076	2.293197429	54979.31921	39035.85912	15943.4601	149.8262912	207.6875991	94.30539328	2152567.164	1506655.658	645911.5059
2011	52.48351366	67.11692961	38.80079947	774695.3481	500029.9032	274665.4448	3.619711611	5.371687635	2.075444318	52062.49719	37169.08778	14893.40942	138.668044	193.1935168	86.28356959	2034808.809	1432174.337	602634.4725
2012	49.33235317	63.39131346	36.16229638	740214.5666	480983.5299	259231.0367	3.343284527	4.976021677	1.89488664	49732.48979	35664.43275	14068.05704	128.4475805	179.8070428	79.04126252	1930475.294	1365444.572	565030.7219
2013	46.01195956	59.44071749	33.40930145	701709.5653	459238.0674	242471.4979	3.062118929	4.569005692	1.718216348	47021.55109	33833.55039	13188.0007	117.7836399	165.7206844	71.62529458	1813094.531	1288569.188	524525.3421
2014	43.16011225	55.99994666	31.08670324	669523.1288	440902.741	228620.3878	2.842461523	4.256212647	1.576936413	45123.7162	32575.74108	12547.97512	109.2925059	154.635775	65.607193	1724611.281	1231769.876	492841.4059
2015	41.42293116	53.81316288	29.75221071	651773.6781	430427.163	221346.5151	2.721047642	4.071419292	1.511896229	44588.61017	32120.1839	12468.42627	104.3005931	147.6717944	62.544425	1683913.231	1202545.566	481367.6654
2016	40.62027786	52.70113613	29.22121562	648281.4696	428059.7171	220221.7525	2.604276738	3.887325126	1.455229672	44077.53841	31633.20717	12444.33124	100.0622218	141.4700529	60.22993088	1652511.913	1178181.448	474330.4654
2017	40.05962786	51.88425604	28.87739107	647841.8463	427445.7598	220396.0865	2.414548385	3.616430238	1.339049829	42120.41344	30278.6982	11841.71524	94.02458129	133.1726301	56.3897319	1582251.374	1130402.558	451848.8164
2018	39.57443452	51.17527391	28.57465967	647980.5338	427261.3959	220719.1379	2.248910355	3.384346354	1.234685137	40357.52705	29087.49739	11270.02967	88.79896093	126.0556606	52.98533933	1518277.588	1087780.202	430497.3862
2019	39.00333582	50.3907028	28.16887979	646239.5841	426104.431	220135.1531	2.10354644	3.176337722	1.147469976	38908.65214	28075.05685	10833.5953	84.07708502	119.4571605	50.07088577	1463417.224	1050222.687	413194.5368
2020	37.88160566	49.00623957	27.2692293	636093.8901	420412.154	215681.7361	2.001196162	3.032063754	1.084458197	38042.51016	27484.69712	10557.81304	80.21124737	114.1676063	47.57522262	1420618.974	1022038.163	398580.8106
2021	36.27988005	46.74879962	26.26990992	617725.642	406432.7763	211292.8657	1.907265974	2.897157372	1.028765267	37331.56823	26981.0202	10350.54804	76.21543447	108.6581882	45.02129236	1375510.112	991182.1032	384328.0093

Table S5 Average Annual Percentage Change of the ASR for TB incidence, mortality, and DALYs in China from 1990 to 2021, categorized by gender

**Item**	**Year**	**Sex**	**AAPC(%，95%CI)**	***t***	***P***
Age-standardized incidence rate	1990-2021				
		Both	-3.33(-3.45,-3.21)	-53.2694	＜0.001
		Male	-3.09(-3.23,-2.94)	-41.297	＜0.001
		Female	-3.73(-3.91,-3.55)	-40.6552	＜0.001
Age-standardized deaths rate	1990-2021				
		Both	-7.28(-7.55,-7.01)	-50.5709	＜0.001

		Male	-6.84(-7.12,-6.56)	-46.203	＜0.001
		Female	-8.26(-8.56,-7.96)	-51.3936	＜0.001
Age-standardized DALYs rate	1990-2021				
		Both	-6.77(-7.01,-6.54)	-55.062	＜0.001
		Male	-6.31(-6.53,-6.10)	-55.6673	＜0.001
		Female	-7.69(-8.19,-7.18)	-28.697	＜0.001

Table S6 Number and ASR prediction of TB incidence, mortality and DALY in China from 2022 to 2046 based on APC model

Year	Incidence				Deaths				Disability-Adjusted Life Years（DALYs）			
	ASR (per 100000)		Number		ASR (per 100000)		Number		ASR (per 100000)		Number	
	Male	Female	Male	Female	Male	Female	Male	Female	Male	Female	Male	Female
2022	47.14	26.44	414310.6303	213159.4655	2.78	0.99	26665.02485	10053.23723	106.66	45.43	980843.6303	383481.6464
2023	46.22	26.04	409001.5751	210919.1706	2.63	0.94	25740.64766	9704.622069	102.37	44	948309.8407	370764.2435
2024	45.29	25.64	403321.7345	208473.3859	2.47	0.89	24747.09794	9324.853225	98.07	42.58	913477.0283	357110.4923
2025	44.66	25.3	401169.2063	207298.3255	2.39	0.86	24539.7622	9247.577404	95.36	41.48	898013.1384	350485.8525
2026	44.03	24.97	398659.5361	206042.3656	2.31	0.83	24291.24483	9152.130042	92.64	40.38	881271.9896	343303.6641
2027	43.4	24.63	395822.7835	204732.0493	2.23	0.79	23987.31324	9032.747102	89.93	39.28	863124.9207	335504.0489
2028	42.77	24.3	392879.5603	203420.6305	2.15	0.76	23640.17292	8893.817949	87.22	38.18	843639.7638	327104.8434
2029	42.14	23.96	389869.5299	202092.6774	2.07	0.73	23251.70791	8736.464327	84.51	37.08	822699.3571	318082.4994
2030	41.84	23.77	390486.2987	202359.6851	2.04	0.72	23472.9908	8822.735623	83.28	36.57	817936.5517	315728.3824
2031	41.53	23.57	390792.4128	202576.8434	2.02	0.71	23668.44564	8898.393427	82.05	36.05	812370.6473	313025.3867
2032	41.22	23.38	390745.5771	202750.4993	1.99	0.7	23824.02954	8957.915239	80.82	35.54	805891.0047	309953.5018
2033	40.92	23.19	389913.8811	202499.9789	1.97	0.69	23951.977	9006.073206	79.59	35.03	798612.9454	306531.7183
2034	40.61	22.99	388653.7668	202028.611	1.94	0.68	24054.45326	9043.745356	78.35	34.52	790452.5821	302733.8933
2035	40.59	22.92	390445.9243	202845.4134	1.96	0.68	24715.42229	9309.404158	78.3	34.48	796136.0022	304633.2992
2036	40.56	22.86	391722.8329	203373.6823	1.97	0.69	25358.36981	9567.842194	78.24	34.43	800964.1463	306138.3272
2037	40.54	22.79	392432.997	203524.0542	1.99	0.69	25965.83315	9811.615477	78.18	34.39	804584.6486	307092.2433
2038	40.51	22.72	392677.2225	203468.5381	2.01	0.69	26544.06141	10046.85431	78.12	34.34	807047.9591	307734.0814
2039	40.49	22.65	392488.8206	203150.5693	2.03	0.7	27096.16653	10273.80982	78.06	34.3	808333.4424	307959.4531
2040	40.48	22.59	392723.711	202813.1096	2.05	0.7	27741.26894	10544.68707	78.03	34.25	810979.3325	308820.3634
2041	40.47	22.52	392672.1083	202291.62	2.07	0.71	28370.56959	10806.00334	78	34.21	812863.0131	309367.9699
2042	40.45	22.45	392426.5247	201588.5925	2.09	0.72	28974.97478	11052.07664	77.97	34.17	814000.0887	309609.5796
2043	40.44	22.38	392073.0889	200831.382	2.11	0.72	29550.98754	11280.79217	77.95	34.12	813992.2262	309378.3217

2044	40.43	22.32	391576.2435	199931.6596	2.13	0.73	30098.1976	11493.59192	77.92	34.08	812736.8838	308647.0763
2045	40.42	22.25	390918.6511	198893.1913	2.15	0.73	30607.69519	11689.60258	77.89	34.04	810420.4916	307495.9329
2046	40.4	22.18	390119.0457	197745.2638	2.18	0.74	31077.26485	11870.03736	77.86	33.99	807378.7445	306044.0004

Table S7 Number and ASR prediction of TB incidence, mortality and DALY in China from 2022 to 2046 based on BAPC model

**Year**	**Incidence**				**Deaths**				**Disability-Adjusted Life Years（DALYs）**			
	**ASR (per 100000)**		**Number**		**ASR (per 100000)**		**Number**		**ASR (per 100000)**		**Number**	
	**Male**	**Female**	**Male**	**Female**	**Male**	**Female**	**Male**	**Female**	**Male**	**Female**	**Male**	**Female**
2022	43.40339788	25.19488211	175151.3626	313057.176	2.810202537	0.978134288	6799.855327	20269.24419	97.25847812	41.72349953	290056.0424	701499.5597
2023	41.35810672	24.45477767	170296.8387	298514.7774	2.678161401	0.928238699	6464.017712	19330.44856	90.61499589	39.42308688	274532.329	654041.4317
2024	39.41301307	23.73982813	165528.1974	284539.8817	2.552307958	0.880685418	6140.662391	18426.23407	84.42769479	37.24914656	259723.1981	609520.6739
2025	37.56325011	23.05107523	160849.1356	271100.9703	2.432537764	0.835274753	5828.501307	17556.07798	78.66126692	35.19009164	245554.5248	567713.0101
2026	35.79926244	22.38732118	156257.8658	258148.7655	2.318308108	0.791894073	5527.221267	16717.33816	73.27948339	33.23531384	231974.1236	528418.9362
2027	34.11633835	21.74642538	151757.565	245686.3844	2.209500188	0.750518736	5237.499674	15911.55847	68.25635576	31.38000277	218985.5449	491543.2919
2028	32.51589212	21.1283595	147352.6296	233739.2372	2.106063203	0.711193011	4959.976203	15139.35416	63.57722596	29.62760279	206627.7403	457022.4383
2029	30.99702852	20.53367833	143053.2811	222317.5152	2.007788566	0.673858307	4694.611469	14400.30178	59.22074892	27.97567631	194899.9211	424744.2538
2030	29.55463576	19.96260485	138861.4242	211388.9724	1.914609185	0.638323233	4440.225809	13694.20593	55.1614175	26.41421968	183739.3563	394540.9938
2031	28.1793507	19.41415833	134776.8431	200900.9081	1.825923752	0.604504345	4196.586118	13017.67894	51.37270387	24.93482044	173102.3476	366254.8144
2032	26.86740311	18.88677174	130803.1997	190852.5902	1.741468037	0.572359424	3963.961921	12370.51769	47.83584291	23.53294082	162980.9477	339801.8961
2033	25.62028683	18.37940328	126938.6338	181266.6215	1.661153019	0.541932925	3742.897642	11752.8581	44.54076243	22.21072967	153399.9575	315131.27
2034	24.43727664	17.89186136	123186.3192	172147.4188	1.584869895	0.513163833	3533.157477	11164.55265	41.47259504	20.96586593	144350.9873	292152.0385
2035	23.31369814	17.42371626	119543.5803	163463.6549	1.512589499	0.485852064	3333.416037	10605.49924	38.61322913	19.79000587	135778.6204	270736.0935
2036	22.24077687	16.97320237	116003.3347	155161.4199	1.443757513	0.459924627	3143.354408	10072.28601	35.94357622	18.67650908	127644.5827	250758.1617
2037	21.21548612	16.53916344	112568.8674	147234.7818	1.378016597	0.435326532	2962.919793	9563.390248	33.4505358	17.62184215	119937.7961	232145.6276
2038	20.23947613	16.12085321	109239.8099	139701.6737	1.315241852	0.412102792	2792.533997	9078.371728	31.12757495	16.62802052	112676.53	214856.0708
2039	19.31235537	15.71800704	106016.8774	132560.251	1.255375729	0.390194886	2631.837694	8616.914852	28.96435285	15.69329999	105850.23	198811.6835
2040	18.43021247	15.32988201	102891.0673	125774.8955	1.198413865	0.369406556	2479.382086	8178.439552	26.94791428	14.81076182	99406.83758	183902.9857
2041	17.58549505	14.95433487	99848.65351	119292.8905	1.143906245	0.349669885	2334.712142	7759.797612	25.06428496	13.97461124	93307.13318	170025.9784
2042	16.77594956	14.5906267	96890.01683	113103.0259	1.0916042	0.330925481	2197.532435	7359.567793	23.30435507	13.18198145	87535.81536	157117.3701
2043	16.00352141	14.23898048	94015.99995	107215.7803	1.041488556	0.313216851	2068.083136	6977.464852	21.66390371	12.43481821	82103.62179	145137.5781
2044	15.26843303	13.89984181	91228.19935	101628.0895	0.993551556	0.296497716	1945.989972	6613.17021	20.13575008	11.73205678	77000.47451	134025.3978
2045	14.56787476	13.57277331	88520.08144	96313.64128	0.947766969	0.280600999	1830.04775	6266.040131	18.71059601	11.0683718	72186.6601	123702.7131
2046	13.89585596	13.25554091	85877.20934	91230.72103	0.903744345	0.265471902	1719.883504	5933.369523	17.37834368	10.4388539	67629.05021	114094.3623
